# Microstimulation of Neurons Distinguishes Neural Contribution to Perception

**DOI:** 10.1371/journal.pbio.0020088

**Published:** 2004-03-16

[Fig pbio-0020088-g001]The brain is an overwhelmingly complex organ packed with billions of nerve cells, performing a myriad of different functions. To decipher the roles of individual neurons in processing sensation or actions, scientists can measure the neural activity of animals that are shown particular objects or perform simple tasks. In this way, neurons are categorized as having preferences, also known as selective responses. These techniques have been particularly helpful in determining, or mapping, preferences of visual areas in the cerebral cortex. For example, some neurons respond to the color of an object, while others respond to the direction that object is moving. What is less well understood, however, is how the brain integrates information from individual neurons for complex processes such as perception and behavior. That is, how does neural activity affect what we see and do?

**Figure pbio-0020088-g001:**
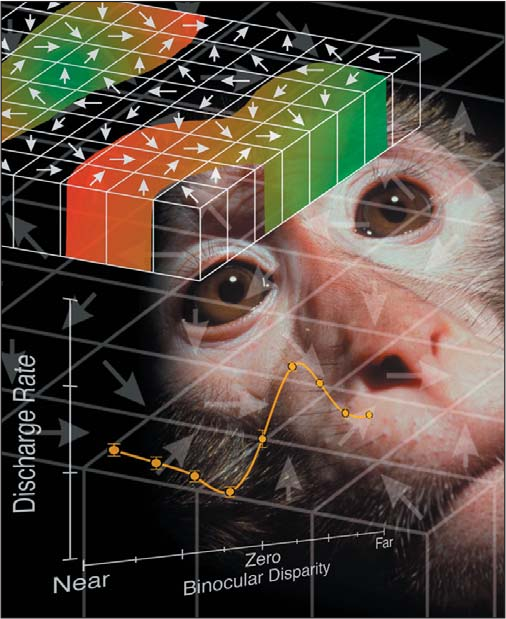
The neural contribution to mokeys' perception of motion

Microstimulation, a technique that activates a cluster of nerve cells by zapping them with a weak electrical current, has helped make causal links between neurons and behavior. For instance, when neurons in an area of the visual cortex that are “tuned” to a particular direction of motion are microstimulated, the way monkeys perceive moving dots on a video screen changes. Microstimulation seems to change what they see. Similar work has also been done for neurons that respond to binocular disparity—the depth-of-field information you gain because each eye has a slightly different view of the world.

But many neurons respond, or are tuned, to more than one dimension, leading scientists to wonder how information from these multidimensional neurons contributes to perception—especially when some of that information is irrelevant to a given task. As they report in this issue, Gregory DeAngelis and William Newsome find that neurons tuned to both direction and binocular disparity contribute little to monkeys' perception of motion.

The researchers asked three rhesus monkeys to determine the direction a group of dots was moving on a TV screen—a task that can be done regardless of the perceived depth of the dots. The authors had already located two different types of neurons in each of the monkey's brains: sites tuned strongly to direction and multidimensional sites tuned to both direction and binocular disparity. They then determined each site's exact preference: the direction of motion and degree of binocular disparity (if present) that triggered maximum neural activity. The researchers then showed the monkeys several sets of video displays, some with the dots moving in the “preferred” direction and some not. The microstimulation acts somewhat like adding dots in the preferred direction—which confuses monkeys when the real dots are moving against preference and aids them in trials of preferred moving dots. If the behavioral effect of microstimulation—whether it be a help or a hindrance—was significant, it meant that the monkeys were monitoring the activated neurons to perform the task at hand; if there was no change, the stimulated neurons were not being recruited.

DeAngelis and Newsome hypothesized that multidimensional neurons (which are also tuned to the irrelevant dimension of binocular disparity) might be ignored during pure motion perception tasks. For two of the three monkeys, this was true. Microstimulation of multidimensional sites had no effect on their behavior, compared to the significant effect of microstimulation of direction-only sites. But for the third monkey, called monkey R, microstimulation of both types of sites had significant effects on his performance. He didn't seem to be ignoring anything. The authors proposed that the monkeys could be using different neural strategies to complete the same task. This conclusion is supported by the fact that monkey R performed better on the task than the other monkeys; he appeared to be recruiting any neuron with applicable information, unlike the others, who seemed to rely on neurons tuned solely to direction of motion. Furthermore, for the few multidimensional sites that affected behavior, their contribution was tempered by how well the depth, or disparity, of the video matched the preference of the stimulated neurons.

The results of this paper show that even if neurons carry information that can aid in perceptual decision making, they may not participate, depending on how they are tuned along other (irrelevant) stimulus dimensions. All directional neurons are not created equal—some are more useful than others for a particular task. Whether neurons that respond to a particular stimulus contribute to the task at hand depends on how closely that stimulus hews to the neurons' preference as well as on the subject's learned strategy for performing the task. This neural flexibility, the authors point out, suggests that the brain uses complex, variable strategies to respond to changing environmental stimuli. Techniques like microstimulation will be helpful in drawing the connections between neural activity and behavior.

